# Arsonic Acid Functional Polymers Enable Stabilization of Iron Oxide Nanoparticles in Aqueous Solution

**DOI:** 10.1002/marc.202500743

**Published:** 2025-12-09

**Authors:** Nhu Thao Huynh, Alexander Rajakanthan, Jurie Tashkandi, Rafia Rafique, Milad Ghorbani, Zihnil A. I. Mazrad, Nicole M. Warne, Kiyonori Suzuki, Karen Alt, Paul Wilson, Kristian Kempe

**Affiliations:** ^1^ Monash Institute of Pharmaceutical Sciences Monash University 381 Royal Parade Parkville Victoria Australia; ^2^ NanoTheranostics Laboratory School of Translational Medicine Monash University Melbourne Victoria Australia; ^3^ Department of Chemistry University of Warwick United Kingdom; ^4^ Department of Material Science and Engineering Monash University Clayton Victoria Australia

**Keywords:** arsonic acid, arsenic, Iron oxide nanoparticle, particle coating

## Abstract

The stabilization of iron oxide nanoparticles (IONPs) with polymer coatings has received immense research interest as it improves their aqueous dispersion and enhances their pharmacokinetics and biodistribution. A range of functional groups including carboxylic acids, silanes, catechols, and phosphonic acids have been incorporated into polymers and investigated as anchoring groups for IONPs surfaces. While some anchors suffer from limitations such as low affinity, poor stability and undesirable reactivity with some substrate surfaces, pentavalent phosphonic acid groups have been shown to be one of the most effective. Here, a new and alternative polymer‐based stabilization strategy for IONPs in aqueous solution, using arsonic acid functional polymers PEG‐*b*‐P(AsAm)‐IONPs has been developed. The arsonic acid groups were shown to provide efficient and stable anchoring to the IONPs surface with transmission electron microscopy (TEM) and dynamic light scattering (DLS) confirming particle colloidal stability in water and in phosphate‐buffered saline over extended periods of time. Importantly, these polymeric scaffolds are cytocompatible with mouse fibroblast cells (NIH/3T3), encouraging their future biomedical applications.

## Graphical abstract

Arsonic acid containing diblock copolymers are developed to incorporate arsonic acid moieties for surface anchoring to IONPs, along with water soluble PEG blocks to impart colloidal stability in aqueous environments. The resulting polymer‐coated IONPs are evaluated for stability in physiological media and cytocompatibility.

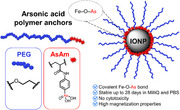

## 1 Introduction

Iron oxide nanoparticles (IONPs) are a class of metal‐oxide nanoparticles that have attracted particular attention due to their superparamagnetic properties. This behavior allows the nanoparticles to be spatially manipulated and located magnetically from a distance, which is desirable for biomedical applications such as drug delivery [1, 2], magnetic hyperthermia [3] and magnetic imaging modalities [4]. The use of IONPs in this context has been advantageous compared to other magnetic nanoparticles due to their facile synthesis, tunable size and structure, and biocompatibility [2]. However, non‐coated IONPs are inherently colloidally unstable in aqueous physiological conditions such that they readily agglomerate, exhibiting ferrimagnetic behavior and become adsorbed with serum proteins, leading to their rapid elimination from blood circulation when administered intravenously [5]. Therefore, the stabilization of IONPs to alleviate these issues has become an active and important research field, with polymer coatings being at the forefront of technology endowing IONPs with enhanced colloidal stability, physicochemical properties and biocompatibility. Moreover, the ability to engineer the surface of IONP with polymers that can contain a variety of functional groups (e.g. for targeting, and therapeutics) can enhance and expand the applications of these hybrid materials [6].

Polymer stabilization of IONPs can be achieved via two main strategies, referred to as “grafting from” and “grafting to” [6]. The “grafting from” strategy involves the initial anchoring of small molecules to the IONP surface, which subsequently initiate or mediate surface‐initiated polymerization directly from the nanoparticle surface. In contrast, the “grafting to” approach employs pre‐synthesized polymers that are functionalized with anchoring groups at the chain end or along the side chain. These polymers are introduced via a ligand exchange reaction, typically replacing small‐molecule ligands (e.g., oleic acid) that initially stabilize the IONPs. While the “grafting from” strategy generally yields a higher grafting density, the “grafting to” approach offers greater control over polymer composition, architecture, and functionality [6].

**SCHEME 1:**
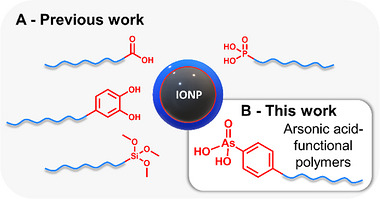
Schematic representation of polymeric ligands used for IONP coatings, (A) previously described functional polymers, (B) arsonic acid functional polymers described in this study.

To date, a variety of functional groups have been shown to graft onto IONPs. Carboxylic acid [7], silane [8], catechol [9] and phosphonic acid [10] functional polymers have all been well studied in the context of stabilizing IONPs (Scheme 1A), however, each of these strategies presents certain limitations. For example, the binding affinity of the carboxylic acid group is relatively weak, limiting their ability to provide long‐term anchoring to the IONP surface [11]. Catechols offer stronger interactions, but, their susceptibility to oxidation at the nanoparticle surface can lead to the formation of quinones, leading to detachment and etching of the particle surface [12]. In recent years, the use of pentavalent acid functional polymers has gained attention. For instance, phosphonic acid‐functionalized polymers stabilize IONPs through Fe─O─P bonds [13], which are stable in aqueous media and at the physiological pH. These bonds are highly resistant to enzymatic cleavage and thermal stress, due to their high bond enthalpy [10, 14]. Additionally, these polymers and polymer‐coated IONPs have revealed minimal cytotoxicity across multiple cell lines, highlighting their potential for biomedical applications [14].

In (bio)chemistry and medicine, arsenic represents a dichotomy between toxicity and function/therapy. From a therapeutic standpoint, it has been used for millennia in Chinese medicine [15], while in the 20th Century (in)organic arsenicals (e.g. Salvarsen and arsenic trioxide) were developed to treat a variety of malignancies [16]. On the other hand, it is a well‐known contaminant of base water supplies, which has been highlighted as a global environmental issue [17] Interestingly, the development of arsenic remediation technologies in water treatment research has shown that iron oxide has a high affinity for arsenic. Consequently, a number of iron oxide‐based adsorbents have been developed to efficiently sequester arsenic from contaminated water [18, 19]. Sitting just below phosphorus in the periodic table, arsenic is also able to form pentavalent arsonic acids. Small organic arsonic acids derived from commercially available *p*‐arsanilic acid have shown to be robust anchors for the surface modification of IONPs compared to carboxylic acid and catechol counterparts [20, 21]. Furthermore, *p*‐arsanilic acid has been simply and efficiently converted into reversible deactivation radical polymerization (RDRP) initiators and monomers for the synthesis of chain‐end and side‐chain arsonic acid functional polymers respectively [22, 23]. Crucially, in all examples to date, arsonic acid functional polymers have been shown to be non‐toxic in vitro, which is essential for application in biomedicine [22, 24].

Herein, we report the first example of IONPs stabilized by arsonic acid‐functionalized polymers (Scheme 1B). To achieve this, diblock copolymers were rationally designed to incorporate arsonic acid moieties for surface anchoring to IONPs, along with water soluble PEG blocks to impart colloidal stability in aqueous environments. In addition to the synthesis and characterization of these polymer‐coated IONPs, their stability in physiologically‐relevant environments and general cytocompatibility was evaluated.

## 2 Experimental Section

### 2.1 Materials

All chemicals were purchased from Sigma–Aldrich and used without further purification unless otherwise stated. 2‐(((Butylthio)carbonothioyl)thio) propionic acid (PABTC) was purchased from Boron Molecular. Dialysis tubing (molecular weight cutoff, MWCO = 1000 g mol^−1^) and Vivaspin Protein Concentrator Spin Columns (MWCO = 100 000 g mol^−1^) were purchased from Repligen and Bio‐Strategy respectively. Oleic acid‐coated iron oxide nanoparticles, OA‐IONP, were provided by Imagion Biosystems. Dulbecco's modified Eagles Medium (DMEM) GlutamaxTM supplemented 1 mm sodium pyruvate 10% v/v fetal bovine serum (FBS), and Alamar Blue were purchased from ThermoFisher Scientific. Mouse embryonic fibroblast cells (NIH/3T3) were purchased from ACC. Deuterated methanol (CD_3_OD) was purchased from Cambridge Isotope Labs. *N*‐(4‐(2,2,3,3,7,7,8,8‐octamethyl‐1,4,6,9‐tetraoxa‐5*λ*5‐arsa‐spiro[4.4]non‐5‐yl)‐phenyl‐2‐propenamide (AsAm(pin)_2_) was synthesized according to literature [25]. *N,N′‐*Dicyclohexylcarbodiimide (DCC), 4‐dimethylaminopyridine (DMAP), 2’2’‐azobisisobutyronitrile (AIBN) and Dulbecco's Phosphate Buffered Saline (PBS) 2’2’2‐trifluroethanol (TFE) were purchased from Sigma‐Aldrich. Toluene, diethyl ether and dichloromethane (DCM) were purchased from Fisher Scientific and used as received.

### 2.2 Instrumentation

#### 2.2.1 Nuclear Magnetic Resonance (NMR)

NMR spectroscopy of all samples was performed using a Bruker AVANCE III HD 400 MHz spectrometer at 25°C running Bruker Topspin Software. Samples were prepared using CD_3_OD. The spectra were processed via MestReNova software.

#### 2.2.2 Size Exclusion Chromatography (SEC)

SEC measurements were conducted using an Agilent 1260 GPC‐MDS fitted with differential refractive index (DRI), light scattering (LS) and viscometry (VS) detectors equipped with 2 × PLgel 5mm mixed‐D columns (300 mm × 7.5 mm), 1 × PLgel 5 mm guard column (50 x 7.5 mm) and autosampler. All samples were passed through a 0.2 µm nylon filter before analysis. The mobile phase was DMF containing 5mM NH_4_BF_4_ with a flow rate of 1.0 mL min^−1^. SEC data was analyzed using Agilent Technologies SEC Software. Calibration curves were produced using Agilent Easi‐Vials linear poly(methyl methacrylate) standards (200–4.7 × 10^5^ g mol^−1^).

#### 2.2.3 Fourier‐Transform Infrared Spectroscopy (FT‐IR)

To analyze chemical bonds, FT‐IR was performed using an FT‐IR spectrometer (Spectrum Two, Perkin Elmer) with a diamond crystal. Spectra were recorded between 4000 and 400 cm^−1^ using the attenuated total reflection (ATR) technique and analyzed using LabSolution IR software.

#### 2.2.4 Transmission Electron Microscopy (TEM)

Transmission electron microscopy (TEM) measurements were performed using a FEI Tecnai G2 T‐20 microscope operated at 200kV accelerating voltage from a lanthanum hexaboride (LaB6) thermal emitter. Samples were prepared in 1 mg mL^−1^ of CHCl_3_ dispersion (as OA‐IONPs) or aqueous dispersions (PEGylated IONPs). A 2 µL aliquot of each sample was pipetted onto a plasma decontaminated (XEI Scientific Evactron 25, 10 s) carbon‐coated copper grid and allowed to absorb for 60 s. Afterward the excess solution was wicked away, and the grid was placed carbon side‐down onto a drop of UranyLess stain for an additional 60 s. Excess stain was wicked away, and the grids were left to air dry before being returned to the sample box for imaging. Images were analyzed using Fiji (v6.5). The reported sizes and histograms were determined by averaging at least 250 particles per sample, reported as Mean ± SD.

#### 2.2.5 Dynamic Light Scattering (DLS)

DLS and Zeta Potential measurements were performed using a Malvern Zetasizer Nano ZS. For hydrodynamic diameters, OA‐IONPs were suspended in CHCl_3_ (1 mg mL^−1^), while polymer‐coated IONPs were suspended in MilliQ/PBS (pH 7.4, 1 mg mL^−1^). Hydrodynamic sizes were reported based on an average of six measurements, and the number of runs was determined automatically. Zeta potential values of the samples were also measured using MilliQ/PBS (pH 7.4, 1 mg mL^−1^). All measurements were carried out at 25°C. All analyte samples were purified by a 0.45 µm nylon filter prior to measurements. Errors were reported corresponding to the standard deviation of the mean.

#### 2.2.6 Thermogravimetric Analysis (TGA)

TGA was performed using a thermal analyzer (TA SDT 650, TA Instruments) at a heating rate of 10°C min^−1^. Approximately, 1‐2 mg of each sample was placed in a platinum pan and heated from 50°C to 800°C under nitrogen purge (50 mL min^−1^). Grafting densities, as chains per nm^2^, were measured based on TGA results and calculated using Equation 1.

**Equation 1**. Mathematic equation to calculate grafting density of P(PEG)‐*b*‐P(AsAm) to IONPs.

$$\begin{array}{ccc} & & Polymer\;Grafting\;Density,\\ & & \;\sigma \;\left(chains\;nm^{-2}\right)=\frac{\left(\frac{wt_{loss}}{M_{n,\;\;\;polymer}}\right)\times N_{A}}{\left(\frac{wt_{0}-wt_{loss}}{m_{IONP}}\right)\times A_{IONP}} \end{array}$$

*where wt_loss_* corresponds to mass loss of polymer coated IONPs determined by TGA. *wt_0_* corresponds to initial mass of polymer coated IONPs determined by TGA. *M*_n_, *_polymer_* corresponds to the molecular weight of the diblock copolymer as determined by ^1^H NMR. *N*_A_ corresponds to Avogadro's constant. *m*_IONP_ corresponds to the mass of a single IONP where the density of Fe_3_O_4_ = 5.17 g cm^−3^. *A_IONP_* corresponds to the area of a single IONP where diameter = 25 nm, as provided by Imagion Biosystems.

#### 2.2.7 Vibrating Sample Magnetometer (VSM)

Magnetization measurements were performed using a Riken BHV‐35H vibrating sample magnetometer (VSM) at 293 K. Dried OA‐IONPs and polymer‐coated IONPs powder samples with a weight of ∼ 5 mg were used. The magnetization was measured under sweeping magnetic field between −15 000 Oe and 15 000 Oe (i.e. µ_0_ *H* = ± 1.5 T) with a sweep rate of 1200 s per loop.

### 2.3 Polymer Synthesis

## 3 General Procedure for PEG Macro‐CTA Synthesis

For PEG_45_‐PABTC, glassware was dried in a 70°C oven for 24 h. Poly(ethylene glycol) methyl ether (40.40 g, 20.11 mmol, *M*_n_ = 2000 g mol^−1^) was dissolved in toluene (500 mL). Approximately 300 mL of toluene was removed by rotary evaporation and DCM was added to afford complete dissolution (100–200 mL). A second solution contained DCC (12.50 g, 60.60 mmol), DMAP (0.25 g, 2.02 mmol) in DCM (10 mL) was added to the PEG solution dropwise under a nitrogen atmosphere. Finally, a solution of PABTC (7.22 g, 30.3 mmol) in DCM (10 mL) was added slowly to the reaction mixture to afford a red solution. The reaction solution was stirred for 16 h at room temperature, and a color changed from red to yellow was observed. The reaction mixture was filtered and then concentrated in vacuo. The crude product was purified by precipitation into cold diethyl ether (∼2 L). The precipitate was collected and the precipitation procedure repeated twice more before being dried under vacuo to yield the macro‐CTA, PEG_45_‐PABTC, as a fine, yellow powder.

^1^H NMR (400 MHz, CDCl_3_, 298K) δ = 4.77 (q, *J*_HH_ = 7.4 Hz, 1H, C**H**CH_3_), 4.23 (t, *J*_HH_ = 7.4 Hz, 2H, SC**H**_2_), 3.72 – 3.45 (m, 183H, OC**H**_2_C**H**_2_), 3.30 (s, 3H, C**H**_3_O), 1.61 (quint, *J*_HH_ = 7.4 Hz, 2H, SC**H**_2_C**H**_2_), 1.54 (d, *J*_HH_ = 7.4 Hz, 3H, CHC**H**_3_), 1.36 (q, *J*_HH_ = 7.4 Hz, 2H, C**H**_2_CH_3_), 0.87 (t, *J*_HH_ = 7.4 Hz, 3H, CH_2_C**H**_3_). ^13^C NMR _APT_ (100 MHz, CDCl_3_, 298K) δ = 59.0 (**C**H_3_O), 48.0 (**C**HCH_3_), 36.9 (S**C**H_2_), 29.9 (SCH_2_ **C**H_2_), 22.1 (**C**H_2_CH_3_), 16.9 (CH**C**H_3_), 13.6 (CH_2_ **C**H_3_). V_max_/cm^−1^: 2882, 2860, 2738, 1737, 1465, 1454, 1360, 1342, 1281, 1237, 1145, 1099, 1062, 948, 841; *M*_n, SEC, THF_ = 2,500 g mol^−1^, Dispersity (*Ð*) = 1.14; λ_max_ = 309 nm.

### 3.1 General Procedure for Synthesis of PEG_n_‐*b*‐P(AsAm)_n_

Exemplarily, for PEG_45_‐P(AsAm)_5_ (2kDP5), AsAm(pin_2_) (3.00 g, 6.62 mmol), PEG_45_‐PABTC (3.31 g, 1.32 mmol) and AIBN (21.7 mg, 0.13 mmol) were dissolved in trifluoroethanol (6.62 mL) to achieve a final monomer concentration of 1 m. The mixture was deoxygenated via N_2_ purging for 15 min and then heated in a sealed vial at 65°C for 300 min. ^1^H NMR_Conv._ = 99%; *M*_n,th_ = 4800 g mol^−1^; *M*_n,SEC_ = 5400 g mol^−1^; *Ð* = 1.06.

To obtain the required AsAm functional group, the pinacol groups were removed from the AsAm(pin_2_) pendant groups via acidic dialysis of the as‐prepared polymer scaffolds. PEG_45_‐P(AsAm)_5_ was dialyzed (MWCO 1000 g mol^−1^) against 0.2 m HCl for 24 h and further dialyzed for 48 h against MilliQ. The dialyzed/deprotected polymer solution was lyophilized to yield PEG_45_‐P(AsAm)_5_ as a white powder. *M*_n,th_ = 3700 g mol^−1^; *M*_n,SEC_ = 4500 g mol^−1^; *Ð* = 1.08. An overview of the polymers prepared can be found in Table 1.

**TABLE 1:** Characterization of PEG‐b‐P(AsAm) used in this work.

Block copolymer	Code	M_n,th_ ^a^ [g mol^−1^]	M_n,SEC_ ^b^ [g mol^−1^]	Ð_m_ ^b^	DP_th_ [AsAm]	DP_calc_ [AsAm]^c^
**PEG_45_‐*b*‐P(AsAm)_5_**	**2kDP5**	3700	4500	1.08	5	4.2
**PEG_45_‐*b*‐P(AsAm)_10_**	**2kDP10**	5200	6700	1.02	10	8.2
**PEG_113_‐*b*‐P(AsAm)_5_**	**5kDP5**	7500	10300	1.06	5	4.3
**PEG_113_‐*b*‐P(AsAm)_10_**	**5kDP10**	9700	12400	1.04	10	8.4

^a^ Calculated from DPs and molar masses of monomers and CTAs.

^b^ Determined by SEC (eluent: DMF + NH_4_BF_4_, Standard: PMMA).

^c^ Actual DPs calculated by ^1^H NMR (CD_3_OD, 400 MHz).

### 3.2 PEGylation of IONPs

Particle coatings were conducted using a ligand exchange reaction, replacing the oleic acid group on the surface of OA‐IONPs with PEG polymers at a weight ratio of 1:3. First, OA‐IONPs (5 mg) were dispersed in CHCl_3_ (5 mg mL^−1^) via sonication for 15 min in an ultrasonic bath (power = 30 W). Polymers (15 mg) in CHCl_3_ (15 mg mL^−1^) were prepared and added drop wise into the OA‐IONPs dispersion. The resulting mixture was vortexed and subsequently sonicated for 15 min. After sonication, the reaction mixture was incubated in an orbital mixer for 24 h at 37°C at 150 rpm. The reaction mixture was then precipitated in an excess of diethyl ether twice. The supernatant was discarded, and the pellet was redispersed in 2 mL of ethanol and 1 mL of ice‐cold petroleum benzine. This solution was exposed to a permanent magnet to isolate polymer‐coated IONPs, followed by the decantation of a clear supernatant. This process was repeated nine times for PEG_113_‐*b*‐P(AsAm)‐coated IONPs to ensure the complete removal of free polymers. Afterward, the excess solvent was removed by airflow, and the polymer‐coated IONPs were redispersed in MilliQ and freeze‐dried for 24 h to obtain a black powder. As for PEG_45_‐*b*‐P(AsAm)‐coated IONPs, membrane centrifugation was applied after the first cycle of magnetic separation. Briefly, the resulting mixture after magnetic separation was redispersed in MilliQ and added into a Vivaspin Protein Concentrator Spin Columns equipped with polyethersulfone membranes (MWCO = 100 000 g mol^−1^). The purification was performed for 10 washing cycles at 2000 rpm for 15 min each. Afterward, the polymer‐coated IONPs were freeze‐dried for 24 h to obtain a black powder.

### 3.3 Cell Culture

Mouse embryonic fibroblast cells, NIH/3T3, used in this study were regularly tested for mycoplasma‐free. Cells between passages 11 and 15 were seeded at a density of 2 × 10^4^ cells cm^−2^ into T25 flasks, 96‐well plates, or 24‐well plates. The NIH/3T3 cells were maintained in DMEM GlutamaxTM supplemented with 1 mm sodium pyruvate, 10% v/v FBS, 100 U mL^−1^ penicillin, and 100 µg mL^−1^ streptomycin. The cells were cultured at 37 °C in a humidified incubator with 5% atmospheric CO_2_. Cell counting for passaging was performed by adding 0.4% Trypan Blue solution to the cells in medium and using a hemocytometer.

### 3.4 In Vitro Cytocompatibility Assays Using Alamarblue Assay

The cytocompatibility of the polymers and polymer‐coated IONPs was tested in NIH/3T3 cells using AlamarBlue assay. Cells were seeded the day before in a black 96‐well plate at a density of 2 × 10^4^ cells per well. Before treatment, the media was removed, and the cells were washed with 1X PBS (pH 7.4) twice. The cells were then incubated with the polymer and polymer‐coated IONPs (diluted in fresh media, prepared through serial dilution) in a humidified incubator with 5% atmospheric CO_2_ at 37°C for 24 h. Samples were run as technical triplicates on the same plate. After incubation, the medium was removed and washed with PBS thrice. Subsequently, a 10% (v/v) solution of AlamarBlue in DMEM was added. The cells were incubated for an additional 4 h at 37°C. Cell viability was determined by measuring the fluorescence (excitation: 540 nm, emission: 590 nm). Wells incubated without cells were used as blank. Control samples were cells incubated with DMEM (without sample). The viability was calculated using equation (Equation 2). The data presented are representative of a minimum of two biological replicates where each sample was run in triplicate. Errors reported correspond to the standard deviation of the mean.

**Equation 2**. Mathematic equation used to calculate cell viability of samples in NIH/3T3 cells.

$$Cell\;viability\;\left[\%\right]=\frac{\left(FI\;sample\;-FI\;blank\right)}{\left(FI\;control\;-FI\;blank\right)\;}\times 100$$

FI corresponds to fluorescence intensity.

## 4 Results and Discussion

**SCHEME 2:**
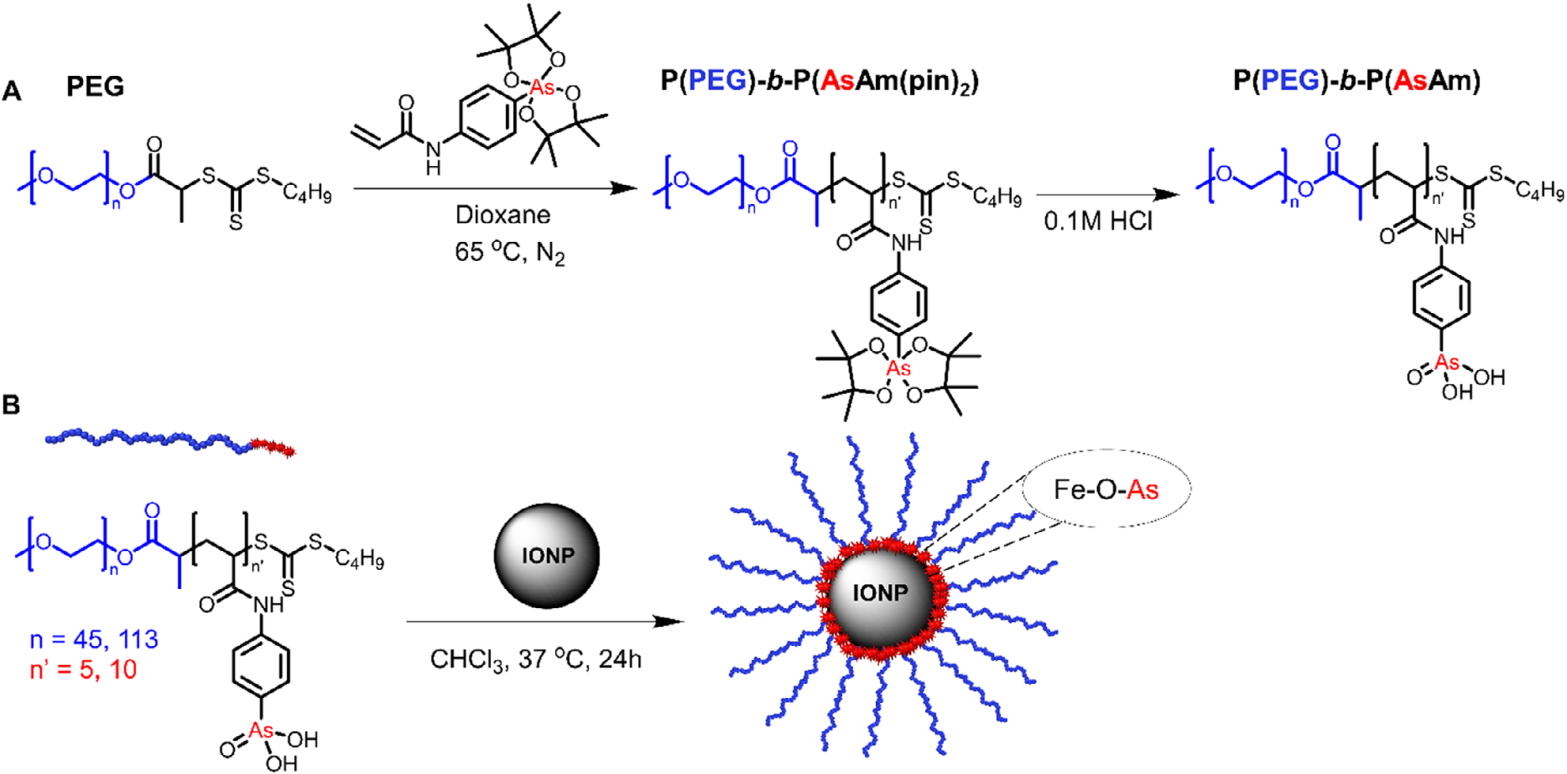
Synthesis approach of arsonic acid functional polymer stabilized IONPs. (A) Synthesis pathway for PEG‐*b*‐P(AsAm). (B) Grafting‐to oleic‐acid stabilized IONP via ligand exchange reaction.

The primary aim of this study was to develop an arsenic‐based polymeric coating system, which can stabilize IONPs in physiologically‐relevant environments. To this end, a library of four diblock copolymers consisting of hydrophilic blocks of linear PEG with varying chain lengths (with degree polymerization (DP) of 45, *M*_n_ = 2000 g mol^−1^; and DP 113, *M*_n_ = 5000 g mol^−1^) as the stabilizing block, and arsonic acid‐containing blocks (AsAm) of varying chain length (DP = 5 and 10) as the anchoring block were synthesized (Scheme 2A). The DPs of AsAm were chosen to be in a range, which was previously shown for phosphonates to lead to efficient IONP functionalization and stabilization [10]. Subsequently, a ligand exchange reaction was employed to graft these polymers onto the surface of IONPs (Scheme 2B). After successful coating, the colloidal stability of polymer‐coated IONPs in MilliQ and PBS was evaluated. The most stable IONPs were chosen for further studies, including magnetic properties characterization using VSM, and cytocompatibility assessment with the Alamarblue assay.

### 4.1 Polymer Synthesis and Characterization

To prepare the different diblock copolymers, two different PEG‐CTAs were prepared by esterification of the respective mPEG‐OH with PABTC. Through RAFT polymerization of the pinacol‐protected arsanilic acid acrylamide monomer, AsAm(pin)_2_, four diblock copolymers were obtained, namely PEG_45_‐*b*‐(AsAm(pin)_2_)_5_, PEG_45_‐*b*‐(AsAm(pin)_2_)_10_, PEG_113_‐*b*‐(AsAm(pin)_2_)_5_ and PEG_113_‐*b*‐(AsAm(pin)_2_)_10_. Subsequently, the protecting groups were removed to yield the target PEG‐*b*‐P(AsAm) diblock copolymers consisting of a water‐soluble PEG block and an arsonic acid‐containing block (Scheme 2A).

**FIGURE 1:**
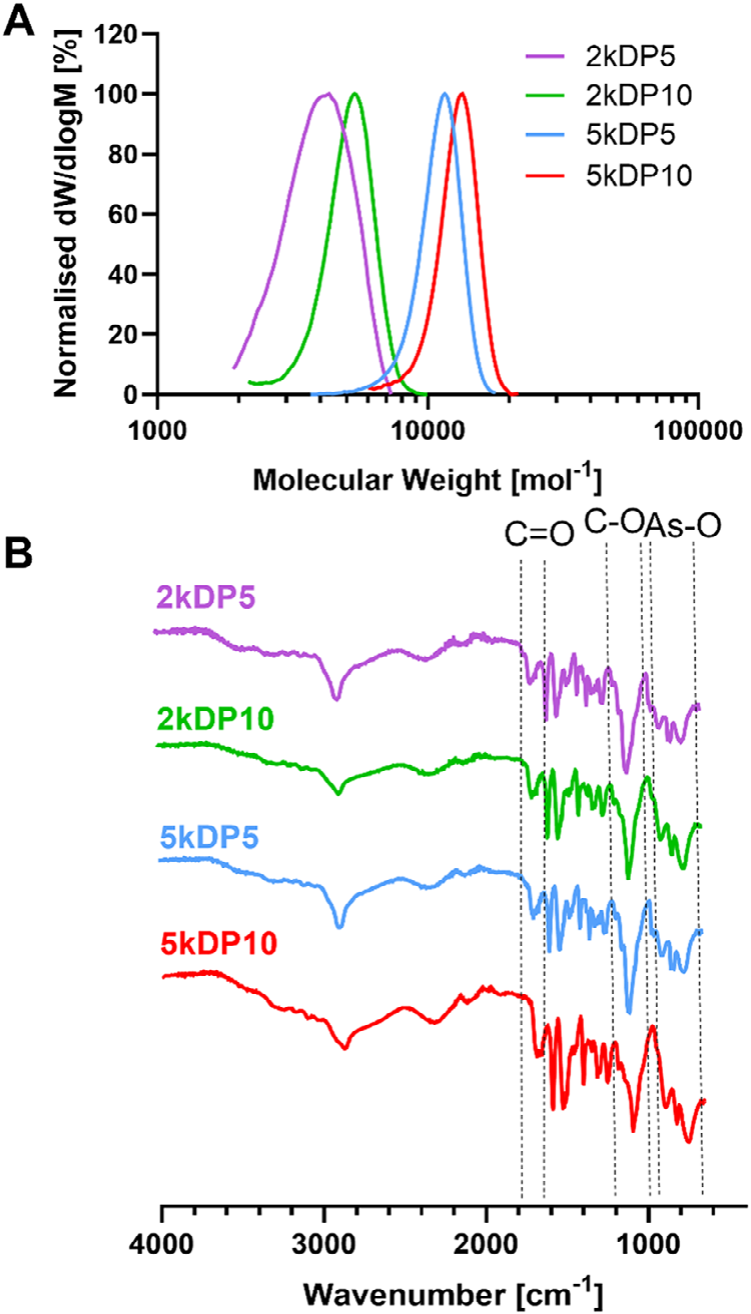
(A) SEC traces of 2kDP5, 2kDP10, 5kDP5 and 5kDP10, showing a shift to higher molecular weight between 2kDP5 and 2kDP10, 5kDP5 and 5kDP10, confirming the different degrees of polymerization. Four polymers were dissolved in DMF with 5 mmol NH_4_BF_4_ as an additive (eluent), 1.0 mL min^−1^ flow rate at 50°C. (B) FT‐IR spectra of 2kDP5, 2kDP10, 5kDP5, and 5kDP10 with characteristic peaks of PEG (C─O) and AsAm (C═O and As─O).

The polymer library was characterized using ^1^H NMR, SEC and FT‐IR spectroscopy. The number of arsonic acid anchoring groups was determined by the integral ratio of the terminal methyl group of PEG and AsAm groups in the ^1^H NMR spectra (Figures S1 and S2 and Table 1). Additionally, SEC was used to evaluate their molecular weight distributions. Well‐defined polymers with *Đ* < 1.20 were prepared (Figure 1A). FT‐IR analysis demonstrated similar spectral features for all four polymers, with characteristic stretching vibrations at 1710 and 1110 cm^−1^ corresponding to the C═O and C─O bonds, respectively. The C═O band is attributed to the amide group of the arsonic acid‐based block, while the C─O stretching originates from the PEG segment. Additionally, As─O bond vibrations were observed in the region from 1000 to 750 cm^−1^ (Figure 1B). Overall, these characterization results confirm the successful incorporation of the functional AsAm segments into the copolymers. Importantly, all four arsonic acid functional polymers were observed to be well soluble in water.

### 4.2 IONP Coating with Arsonic Acid Functional Copolymers

#### 4.2.1 IONPs Coating and Purification

It was hypothesized that the arsonic acid block would promote efficient and stable modification of IONP surfaces through covalent Fe─O─As bond formation, which possess a high bond enthalpy [20, 21]. To graft PEG‐*b*‐P(AsAm) onto IONPs surfaces, OA‐IONPs (25 nm) were mixed with PEG‐*b*‐P(AsAm) to allow for a ligand exchange reaction between the oleic acid and arsonic acid functional polymers at 37°C (Scheme 2B). Although this method is particularly effective in producing highly stable and uniform nanoparticles with well‐controlled hydrodynamic sizes, the primary challenge lies in the complete removal of residual components after the reaction, including ligands, excess polymers, and unreacted NPs [26]. To isolate polymer‐coated IONPs, three purification techniques were explored: (i) precipitation, (ii) magnetic decantation and (iii) membrane centrifugation (Figure S3). Owing to differences in chain length between PEG_45_ and PEG_113_, different purification approaches were applied. For PEG_113_‐b‐P(AsAm)‐coated IONPs, magnetic decantation was sufficient to achieve effective purification. In contrast, this technique was ineffective for PEG_45_‐*b*‐P(AsAm) coated IONPs, likely due to increased flexibility and steric stabilization by the shorter PEG (DP 45) chains, leading to more stable colloidal suspension and thereby resistance to magnetic separation [27]. Consequently, membrane centrifugation was employed as an alternative purification approach for PEG_45_‐*b*‐P(AsAm) coated IONPs.

First, all PEG‐*b*‐P(AsAm) coated IONPs were precipitated in diethyl ether, effectively removing hydrophobic components, such as oleic acid, chloroform and unreacted nanoparticles. This step also helped eliminate insufficiently coated IONPs. Next, the precipitated IONPs were resuspended and subjected to magnetic decantation in a solvent mixture of ethanol: petroleum benzine (2:1, v/v). This step ensured the complete removal of excess polymers after the reaction. PEG_45_‐*b*‐P(AsAm) coated IONPs exhibited poor separation efficiency, requiring an extended period of time for a single magnetic separation. Therefore, these particles were further purified by membrane purification. In contrast, PEG_113_‐b‐P(AsAm)‐coated IONPs were magnetically separated from the ethanol: petroleum benzine mixture within seconds (for 5kDP5‐IONPs) or minutes (for 5kDP10‐IONPs). This washing step was repeated nine times to remove all excess polymer. According to a previous study, three magnetic decantation–resuspension cycles are sufficient to effectively eliminate excess polymer, indicating that our nine‐cycle washing ensured the purity of the IONPs [26].

#### 4.2.2 Colloidal Stability Study in MilliQ and PBS

The ligand exchange reaction yielded four distinct polymer‐coated IONPs: 2kDP5‐IONPs, 2kDP10‐IONPs, 5kDP5‐IONPs and 5kDP10‐IONPs. Successful surface functionalization and purification were confirmed by dynamic light scattering (DLS), which showed single, monomodal size distributions for all samples in MilliQ (Figure S4). To examine the colloidal stability, the hydrodynamic diameter of the IONPs were monitored over the course of 14 days of storage in MilliQ (Figure S5). Both 5kDP5‐IONPs and 5kDP10‐IONPs were relatively unchanged with low PDI values (< 0.2) (Figure S5 and Table S1), indicative of excellent colloidal stability. In contrast, 2kDP5‐IONPs and 2kDP10‐IONPs showed clear signs of aggregation with increasing hydrodynamic sizes and PDI values (> 0.2) over time (Figure S5 and Table S1). These findings were further supported by the visual observation of sedimentation in 2kDP5‐IONP dispersions. Moreover, poor dispersibility of 2kDP5‐IONPs and 2kDP10‐IONPs in PBS was observed (Figure S6). Thus, it was concluded that the PEG_45_‐based systems are insufficient for effective stabilization of IONPs in aqueous media. This observation is consistent with previous reports indicating that PEG_45_ (2000 g mol^−1^) is less effective than PEG_113_ (5000 g mol^−1^) in stabilizing nanoparticles under physiological conditions [25, 28, 29]. The enhanced performance of PEG_113_ (5000 g mol^−1^) is attributed to the increased steric repulsion imparted by longer polymer chains, which reduces interparticle interactions and aggregation [30]. Consequently, long‐term stability evaluations in both MilliQ and PBS were conducted only for 5kDP5‐IONPs and 5kDP10‐IONPs.

**FIGURE 2:**
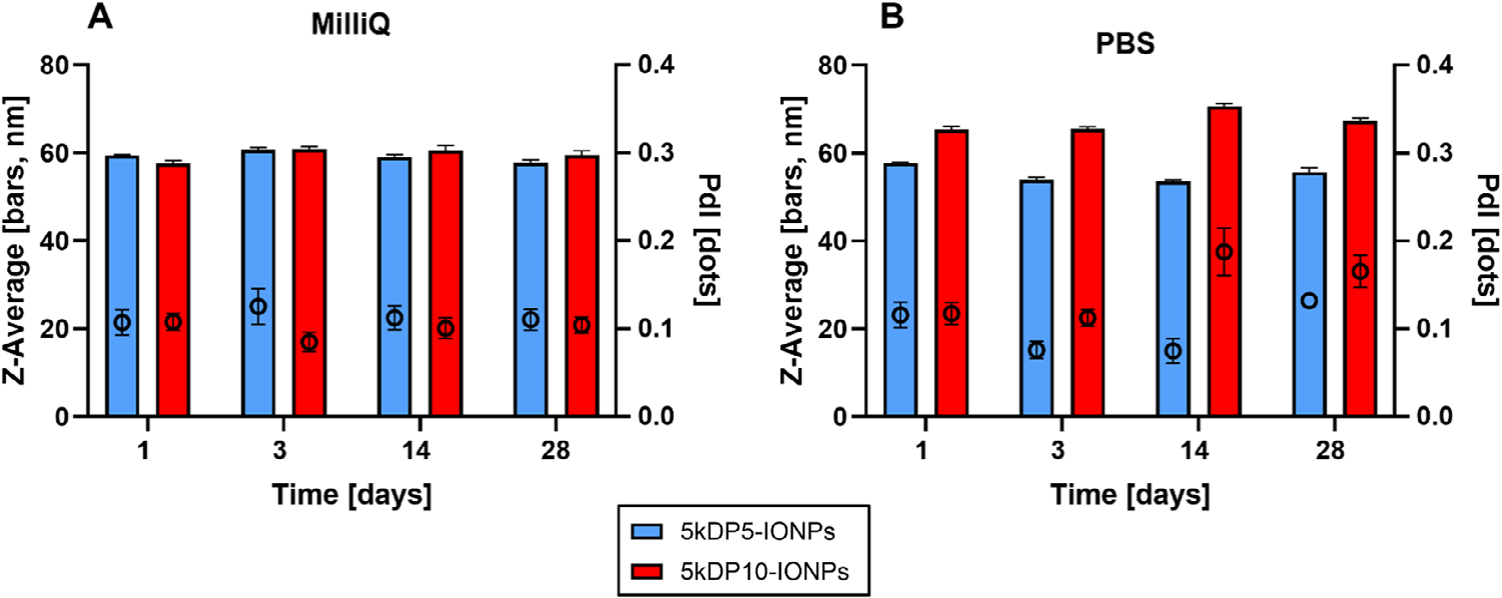
The colloidal stability study of 5kDP5‐IONPs and 5kDP10‐IONPs (1 mg mL^−1^in MilliQ and PBS), as measured by DLS. Z‐Average and PDI values (A) in MilliQ, and (B) in PBS.

The stability analysis of 5kDP5‐IONPs and 5kDP10‐IONPs was further assessed in MilliQ and PBS over a 28‐day period. After 28 days, no visible sedimentation of IONPs was observed in both media, indicating sustained dispersion. DLS measurements of the particle dispersions in MilliQ and PBS (Figure 2) confirmed these observations, showing consistent hydrodynamic diameters and PDI values over the entire study period. These results demonstrate that both PEG_113_‐b‐P(AsAm) coatings effectively maintain nanoparticle stability under aqueous and physiologically relevant conditions. The observed colloidal stability is indicative of strong and persistent binding of arsonic acid moieties to the IONP surface, with no significant evidence of hydrolysis or ligand displacement over time [31].

#### 4.2.3 FT‐IR Spectroscopy, TEM, and Zeta Potential

FT‐IR spectra of 5kDP5, OA‐IONPs, and 5kDP5‐IONPs were analyzed to confirm the formation of covalent Fe─O─As bonds, explaining the strong interactions between arsonic acid moiety and the IONP surface (Figure S7). The spectrum of 5kDP5‐IONPs revealed a combination of characteristic peaks of both 5kDP5 and OA‐IONPs. Specifically, the characteristic Fe─O stretching vibration at 670 cm^−1^ is present in the 5kDP5‐IONPs spectrum, mirroring the profile of OA‐IONPs, where the characteristic peak of Fe─O from iron oxide nanoparticles is widely known to appear in the range of 700–425 cm^−1^ [32]. Moreover, the signal at 836 cm^−1^ corresponds to the As─O─Fe bond formed between an arsonic acid and the hydroxyl groups of iron oxide nanoparticles, confirming the successful conjugation of arsonic acid functionalities onto the surface of IONPs [21, 22, 33, 34]. Furthermore, a strong band at 1116 cm^−1^, corresponding to C─O stretching vibrations characteristic of the PEG backbone appeared [32]. These spectral changes are consistent with the successful ligand exchange and covalent attachment of the 5kDP5 polymer to the IONP surface.

**FIGURE 3:**
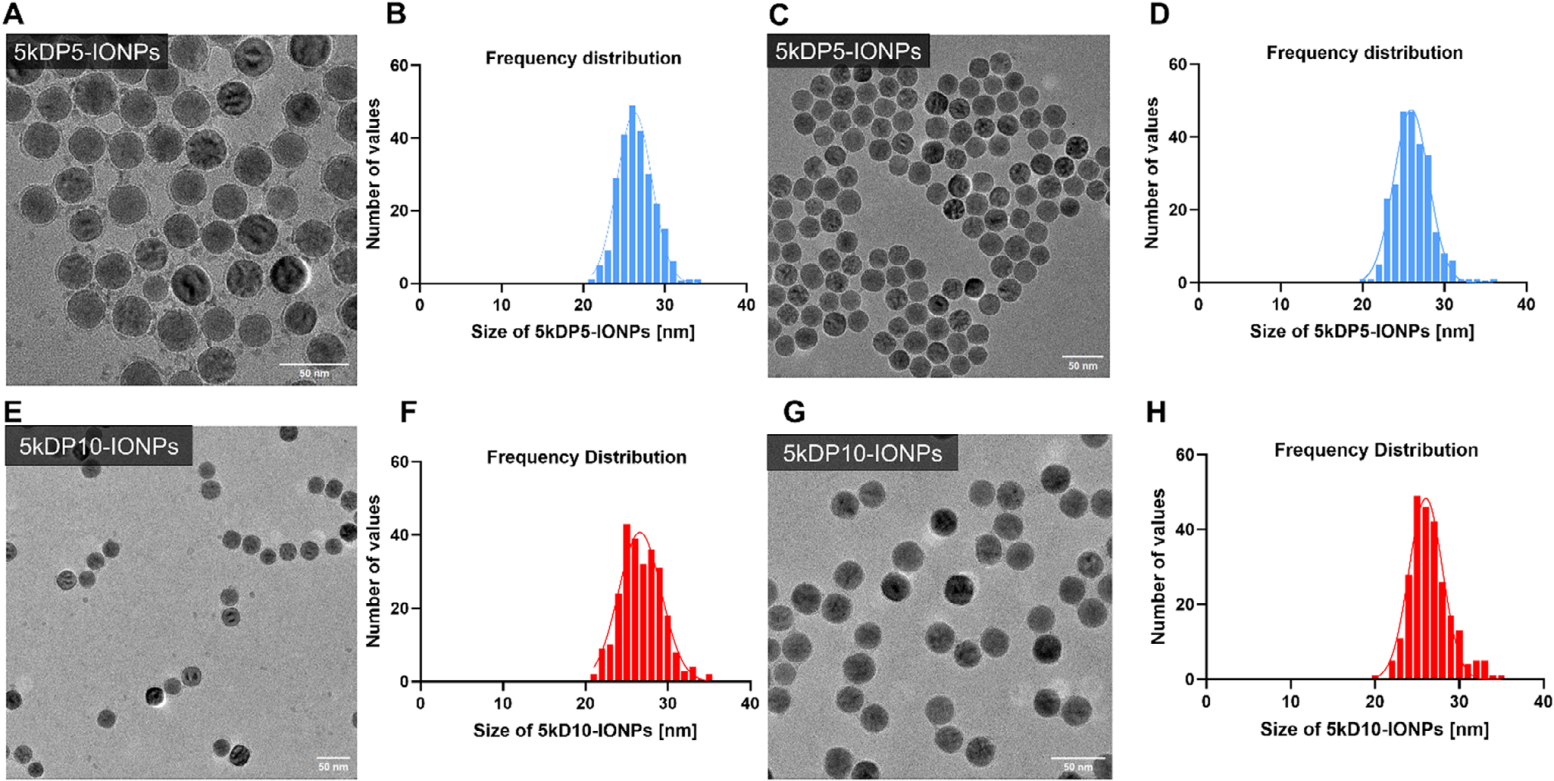
TEM micrographs of 5kDP5‐IONPs (A‐D) and 5kDP10‐IONPs (E‐H) on day 1 (A/B, E/F) and day 28 (C/D, G/H). Corresponding size distribution histograms (B, D, F, H). Scale bar = 50 nm.

TEM analysis was conducted before and after storage in MilliQ in 28 days. TEM images of 5kDP5‐IONPs and 5kDP10‐IONPs are shown in Figure 3, revealing a core size of ∼ 27 nm (Table S2) with a spherical shape and a narrow distribution for both samples. Due to the negligible contrast under electron microscopy as compared to the dominant electron density of IONPs, the physical morphology and size distribution are representative of IONPs cores only [35, 36]. Although the polymer coating cannot be directly measured, the narrow size distribution of nanoparticles shown in TEM images (Figure 3B,D,F,H) indicates effective stabilization of IONPs by both 5kDP5 and 5kDP10 polymers, which prevent aggregation upon ligand exchange with oleic acid. After 28 days of storage in MilliQ, the nanoparticles showed no signs of aggregation, retaining their morphology and narrow size distribution, confirming their excellent colloidal stability (Figure 3C,G; Table S2).

Zeta potential measurements showed negative zeta potential values of ∼ ‐15.0 and−25.2 mV for 5kDP5‐IONPs and 5kDP10‐IONPs, respectively (Table S2). These results confirmed the presence of arsonic acid units (negatively charged), which can be either anchored to the surface of IONPs or freely interact with the surrounding environment. The higher negative surface charge of 5kDP10‐IONPs suggests that there are a higher number of free arsonic acid groups than in 5kDP5‐IONPs.

#### 4.2.4 TGA and Grafting Density

**FIGURE 4:**
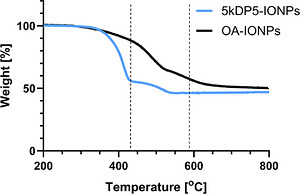
Thermogravimetric analysis (TGA) of OA‐IONPs and 5kDP5‐IONPs.

Successful IONP coating was also confirmed using thermogravimetric analysis (TGA). TGA allows for measuring the grafting density on the surface of IONPs [37]. The percentages of 5kDP5 and 5kDP10 coatings on the surface of IONPs were calculated using TGA data (Figure 4; Figure S8 and Table 2). A weight loss of 43.95% and 37.67% from 200°C to 430°C was observed for the 5kDP5 and 5kDP10 polymer coating, respectively. However, both coatings were not saturated, as evidenced by an additional weight loss of 9.62% and 18.54% for 5kDP5‐IONPs and 5kDP10‐IONPs, respectively, which can be attributed to residual oleic acid. The grafting density of 5kDP5 was calculated to be 1.22 chains nm^−2^, which is deemed to provide sufficient colloidal stability during blood circulation based on previous reports [26, 30]. The lower polymer content of 5kDP10‐IONPs led to a lower grafting density (0.73 chains nm^−2^) as compared to 5kDP5‐IONPs. Importantly, as calculated from the measured grafting densities, both 5kDP5 and 5kDP10 coatings are likely to adopt a “brush” conformation instead of a collapsed “mushroom” conformation, forming a dense layer on the IONPs surface (Table S3). This conformation is expected to impart stealth effects, which are advantageous for prolonging the circulation time in the bloodstream due to the reduction in immune system recognition [38].

**TABLE 2:** Weight loss values and grafting densities determined by TGA analysis.

Sample	Weight loss attributed to Arsonic acid ligand [%]	Weight loss attributed to OA [%]	Total weight remained (IONP core) [%]	Grafting density [chains nm^−2^]
**5kDP5‐IONPs**	43.95	9.62	46.96	1.22
**5kDP10‐IONPs**	37.67	18.54	46.65	0.73
**OA‐IONPs**	—	49.05	50.17	—

### 4.3 Magnetic Properties

**FIGURE 5:**
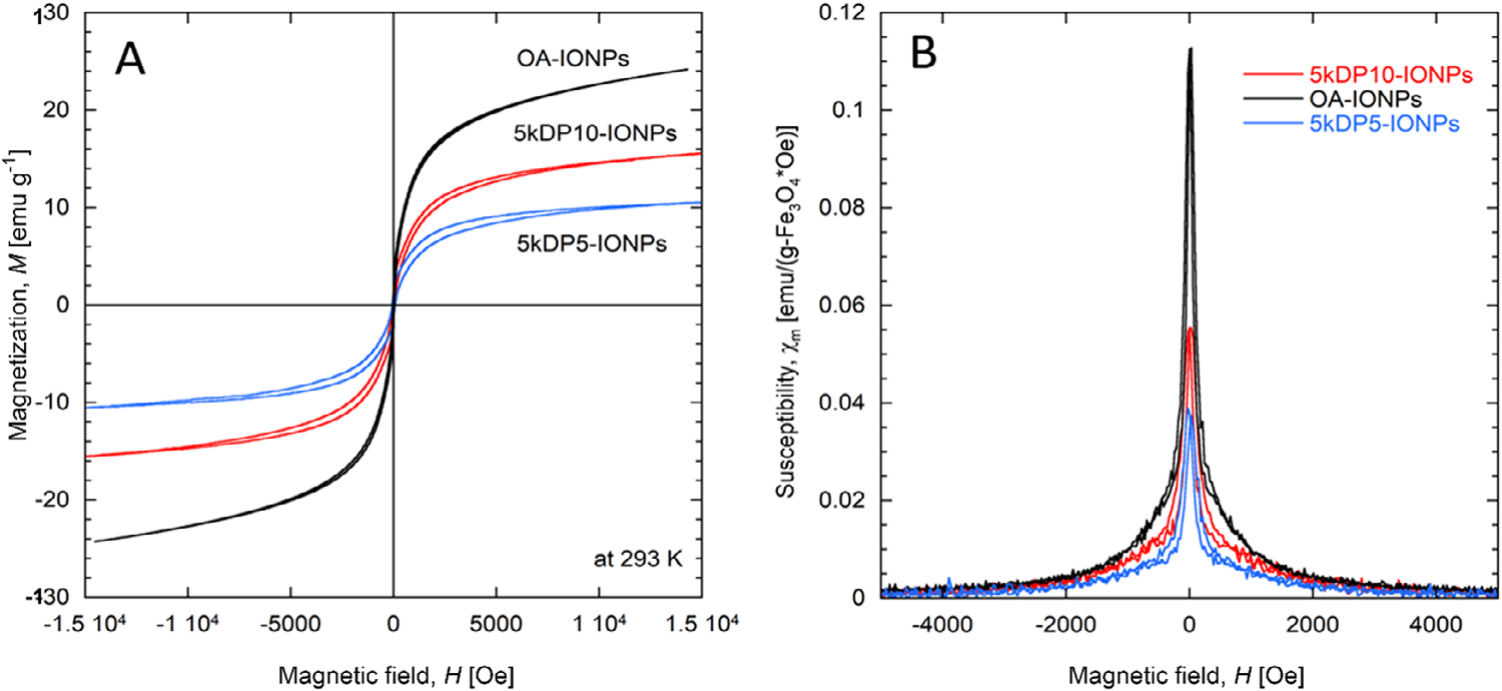
Magnetization (*M‐H*) curves of iron oxide nanoparticles. (A) Hysteresis loop curves of OA‐IONPs (black), 5kDP5‐IONPs (blue) and 5kDP10‐IONPs (red) obtained by VSM at 293K. (B) Corresponding susceptibility curve of OA‐IONPs (black), 5kDP5‐IONPs (blue) and 5kDP10‐IONPs (red).

The magnetization (*M‐H*) curves of the IONPs before and after addition of the arsenic acid functional polymers were measured on a Vibrating Sample Magnetometer (VSM) (Figure 5A). All three *M‐H* curves exhibit relatively high susceptibility even at the near‐saturation region, reflecting the characteristic behavior of superparamagnetic particles. The measured saturation magnetization, defined by the *M* value at µ_0_ *H* = 1.5 T, normalized by the sample mass were found to be 24.3 emu g^−1^ for OA‐IONPs, 10.5 emu g^−1^ for 5kDP5‐IONPs and 15.6 emu g^−1^ for 5kDP10‐IONPs. The polymer‐coated IONPs showed lower saturation magnetization values than OA‐IONPs, confirming the replacement of oleic acid with bulky, non‐magnetic polymer coatings on the surface of IONPs. Noticeably, the decrease in magnetization is influenced by the grafting density. 5kDP5‐IONPs, with a higher grafting density of 1.22 chains nm^−2^, has lower magnetization as compared to 5kDP10‐IONPs with a grafting density of 0.73 chains nm^−2^. Using the TGA results in Table 2, the saturation magnetization value normalized by the mass of IONP core was estimated. The resultant values for OA‐IONPs, 5kDP5‐IONPs and 5kDP10‐IONPs are 48.3, 22.4, and 33.3 emu g_Fe3O4_^−1^, respectively. These values suggest that the polymer layer, to some extent, exhibited interference with the intrinsic magnetic properties of the iron oxide core, aligning well with previous studies [39, 40]. Despite the decrease in magnetization, these values maintain within the optimal range for future magnetic imaging applications (20–80 emu g_Fe3O4_^−1^) [41].

Moreover, the M–H loops in Figure 5A exhibit increased hysteresis after surface modification, indicating that the surface‐modified superparamagnetic particles are partially magnetically blocked at room temperature. However, the remanence ratio (Mr/Ms) remains low, at approximately 0.1, which is significantly smaller than that of magnetically ordered particles (typically 0.5–0.8, depending on anisotropy symmetry). This suggests that the IONPs remain predominantly unblocked [42]. Figure 5B illustrates the susceptibility (d*M*/d*H*) of the three samples, which all display a sharp peak near zero magnetic field. This behavior indicates that all nanoparticles can rapidly respond to the application or removal of an external magnetic field, further supporting their potential as negative contrast agents [40, 43]. The peak mass susceptibility observed in Figure 5B is comparable to reported literature values [40].

### 4.4 Cell Toxicity Assays

An AlamarBlue assay was performed to evaluate the cytotoxicity of arsonic acid functionalized polymers (5kDP5 and 5kDP10) and the coated IONPs (5kDP5‐IONPs and 5kDP10‐IONPs) against mouse fibroblast NIH/3T3 cells. Cytotoxicity of the polymers was assessed over the concentration range of 0.03125–2 mg mL^−1^. The polymer‐coated IONPs were tested across the same concentration range based on the IONPs content (i.e., 0.03125–2 mg mL^−1^ of iron oxide core) according to TGA data.

**FIGURE 6:**
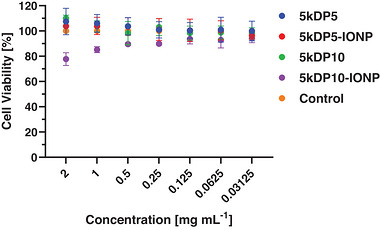
In vitro cytocompatibility of NIH/3T3 cells when treated with arsonic acid functional polymers (5kDP5 and 5kDP10) and their grafted IONPs. Cells were exposed to a range of concentrations from 2 to 0.03125 mg mL^−1^ for 24 h. Control samples were incubated with DMEM (without polymer). Data represents two biological replicates, with each replicate consisting of three technical repeats (*n* = 3). Data are reported as Mean ± SD (*n* = 3).

Neither polymer impacted cell viability (up to 2 mg mL^−1^), highlighting excellent biocompatibility. This is also consistent with our recent findings on arsonic acid functional polymers, which showed non‐toxic profiles against NIH/3T3 cells treated at concentrations up to 8 mg mL^−1^ [22, 44, 45]. Our results further confirmed that changing the local environment with arsenic atoms does not alter the magnitude of cell viability. Regarding polymer‐coated IONPs, 5kDP5‐IONPs showed no significant cytotoxicity even at the highest concentration, with the cell viability level consistently at ∼100% (Figure 6). In contrast, 5kDP10‐IONPs resulted in a reduction (∼80%) of cell viability against NIH/3T3 cells for the highest concentration tested. It is important to note that at this concentration, sedimentation of the 5kDP10‐IONPs in cell culture media was observed. This agglomeration might be attributed to their lower colloidal stability in media culture stemming from a lower grafting density as compared to 5kDP5‐IONPs (0.73 vs. 1.22 chain per nm^−2^).

## 5 Conclusions

In summary, we have reported the synthesis of well‐defined block copolymers consisting of a hydrophilic PEG block, for IONP stabilization, and an arsonic acid functionalized block, for stable anchoring to IONP surfaces. The block copolymer scaffolds have been shown to be cytocompatible with mouse fibroblast cells (NIH/3T3) and undergo ligand exchange reaction with OA‐IONPs to yield PEG‐*b*‐P(AsAm)‐IONPs with surface coverages in the range of 0.73–1.22 chains nm^−2^. The Fe─O─As bonds formed upon ligand exchange exhibit excellent stability in an aqueous environment over extended time scales (up to 28 days). While the polymer‐coated IONPs exhibited lower magnetization values than OA‐IONPs, hysteresis loops showed that both 5kDP5‐IONPs and 5kDP10‐IONPs exhibited low remanent magnetization and susceptibility (dM/dH) measurements indicated that the polymer coated IONPs could rapidly respond at an external magnetic field further indicating retention of magnetic properties. Overall, we have successfully reported the functionalization of IONP surfaces with a novel polymeric system, which yield particles with significantly increased colloidal stability, and superparamagnetic potential making polymer functionalized IONPs highly suitable for diagnostic and imaging purposes in the future.

## Author Contributions

The manuscript was written through the contributions of all authors.

## Conflicts of Interest

The authors declare no conflicts of interest.

## Supporting information




**Supporting File**: marc70167‐sup‐0001‐SuppMat.pdf

## Data Availability

The data that support the findings of this study are available from the corresponding author upon reasonable request.
