# Supplementary material for: Arsonic Acid Functional Polymers Enable Stabilization of Iron Oxide Nanoparticles in Aqueous Solution
Source: Macromol Rapid Commun. 2025 Dec 9;47(14):e00743. doi: 10.1002/marc.202500743 (PMC13384793; doi:10.1002/marc.202500743)
Supplement: Supplementary file 1 — Supporting File: marc70167‐sup‐0001‐SuppMat.pdf [file MARC-47-e00743-s001.pdf]

Supporting Information

Arsonic acid functional polymers enable stabilization of iron oxide nanoparticles in aqueous solution

Nhu Thao Huynh,[a#] Alexander Rajakanthan,[a#] Jurie Tashkandi,[b] Rafia Rafique,[c] Milad Ghorbani,[a,d] Zihnil A. I. Mazrad,[a] Nicole M. Warne,[a] Kiyonori Suzuki,[d] Karen Alt,[b] Paul Wilson,[c] and Kristian Kempe [a,d]**

[a] Monash Institute of Pharmaceutical Sciences, Monash University, 381 Royal Parade, Parkville VIC 3052, Australia

[b] NanoTheranostics Laboratory, School of Translational Medicine, Monash University, Melbourne, VIC 3004, Australia

[c] Department of Chemistry, University of Warwick, CV4 7AL, United Kingdom

[d] Department of Material Science and Engineering, Monash University, Clayton, VIC 3800, Australia

Emails: kristian.kempe@monash.edu; p.wilson.1@warwick.ac.uk; karen.alt@monash.edu

FIGURES

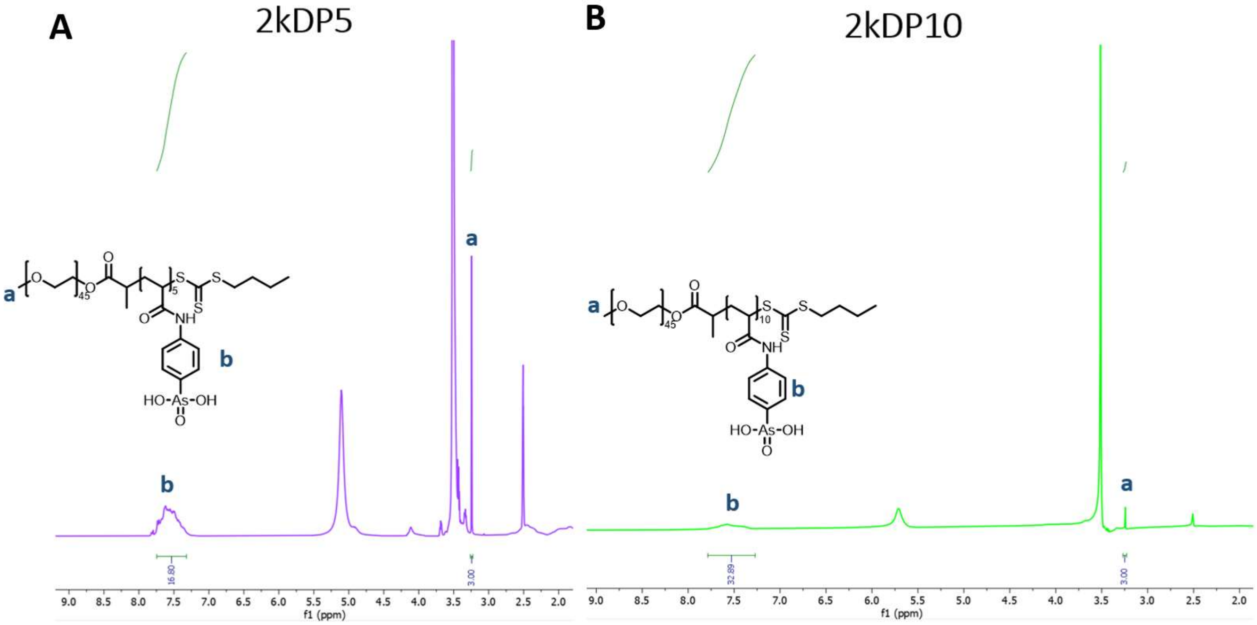

Figure S1. A) ^{1}H NMR (400 MHz, CD_3OD) of 2kDP5, confirming the successful chain extension with ~ 5.6 AsAm monomers. **B)** ^{1}H NMR (400 MHz, CD_3OD) of 2kDP10, confirming the successful chain extension with ~ 10.96 AsAm monomers.

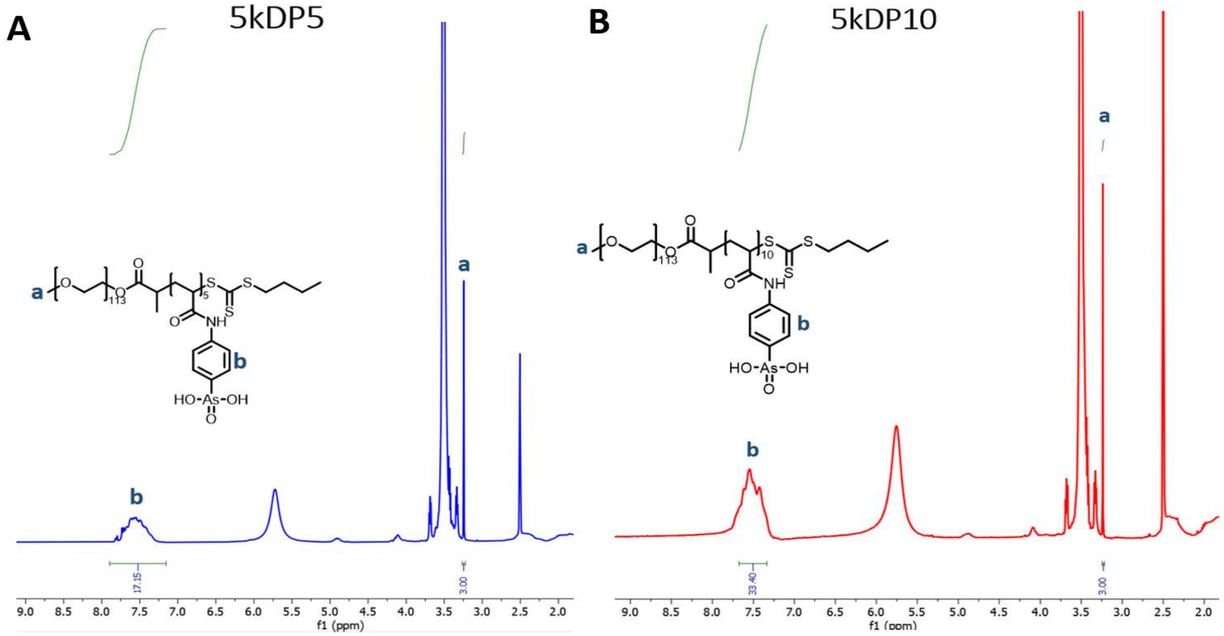

Figure S2. A) ^{1}H NMR (400 MHz, CD_3OD) of 5kDP5 confirming the successful chain extension with ~ 5.7 AsAm monomers. **B)** ^{1}H NMR (400 MHz, CD_3OD) of 5kDP10 confirming the successful chain extension with ~11.1 AsAm monomers.

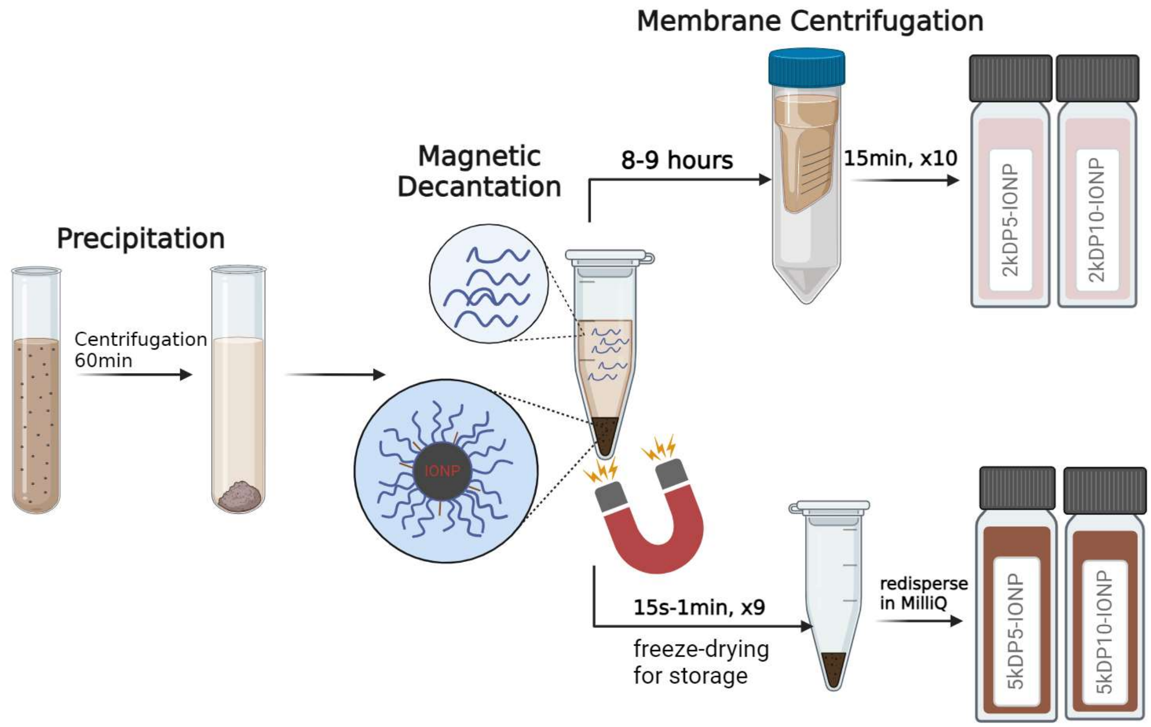

Figure S3. Purification procedure of ligand exchange reaction. 2kDP5 and 2kDP10-coated IONPs underwent three purification steps: precipitation, magnetic decantation and membrane centrifugation. 5kDP5 and 5kDP10-coated IONPs were purified only by precipitation and magnetic decantation.

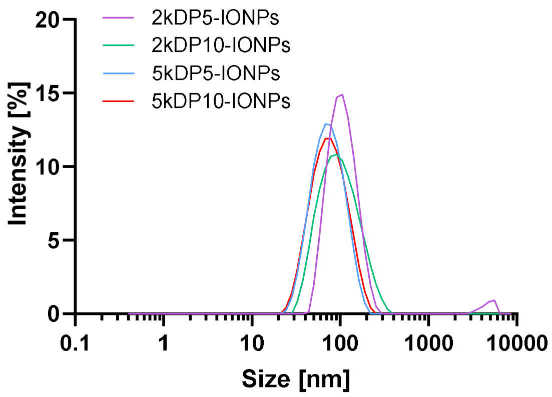

Figure S4. DLS measurements of four polymer-coated IONPs. After purification, the total amount of each sample was freeze-dried and redispersed in 4 ml of MilliQ prior to DLS measurement.

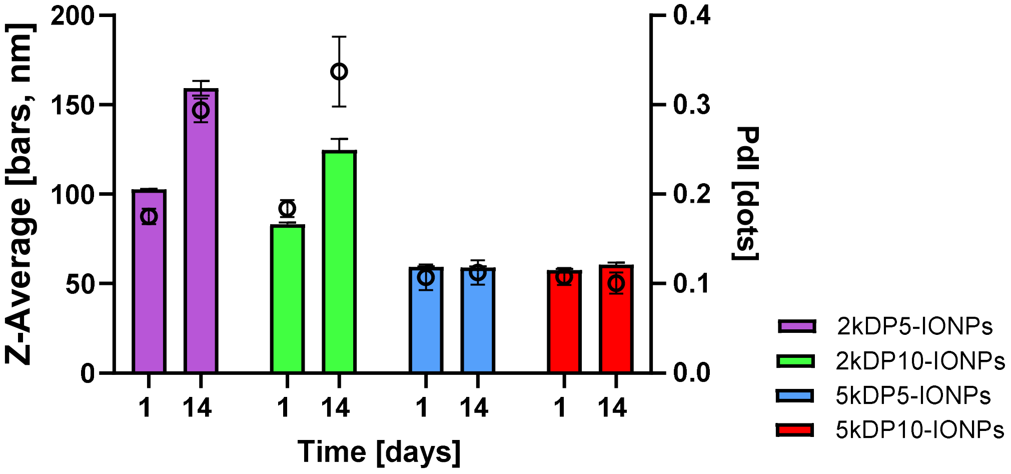

Figure S5. Stability of four polymer-coated IONPs in MilliQ after 14 days. Z-Average and PdI were recorded by DLS with six measurements. All four polymer-coated IONPs after being freeze-dried were redispersed in 4ml of MilliQ and stored for 14 days for preliminarily screening of their stability.

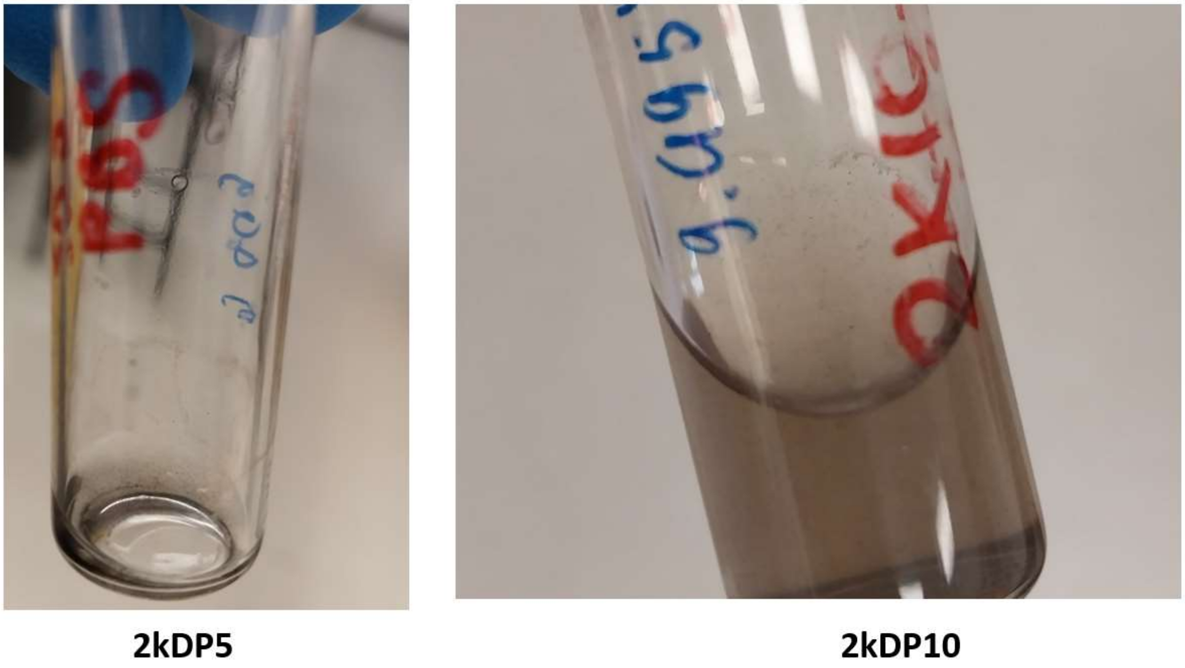

Figure S6. Unstable dispersion of 2kDP5-IONP and 2kDP10-IONP in PBS.

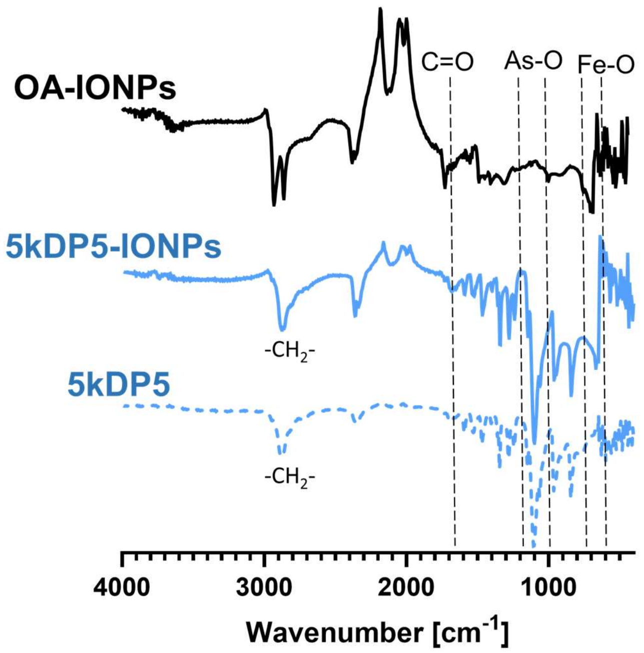

Figure S7. FT-IR spectra of OA-IONP (black line), 5kDP5 (blue line) and 5kDP5-IONPs (fragmented blue line).

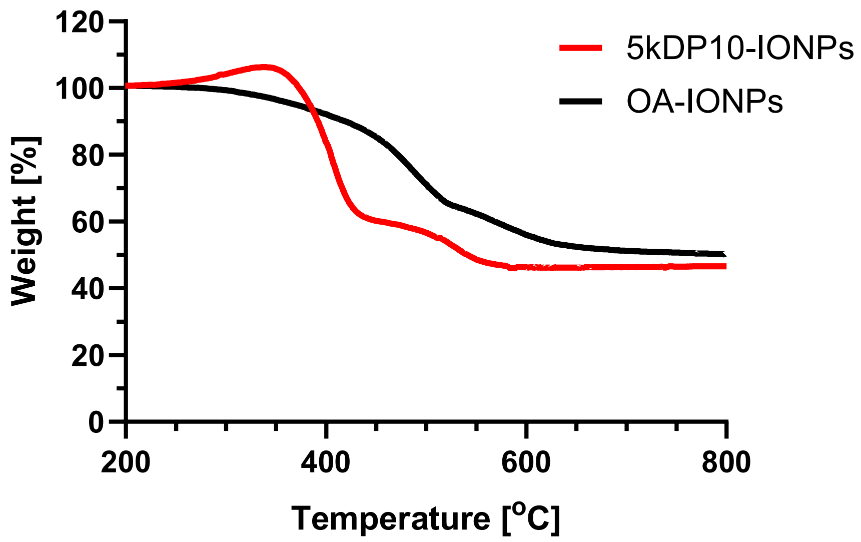

Figure S8. Thermogravimetric analysis of OA-IONPs and 5kDP10-coated IONPs.

TABLES

Table S1. Stability results of IONPs coated by four diblock polymers

PEGylated IONP	Day 1		Day 3		14 days	
	d_{MilliQ} [nm]	PdI	d_{MilliQ} [nm]	PdI	d_{MilliQ} [nm]	PdI
**2kDP5-IONP**	102.67 ± 0.497	0.18 ± 0.01	131.90 ± 1.86	0.26 ± 0.02	159.22 ± 4.18	0.29 ± 0.01
**2kDP10-IONP**	83.32 ± 1.01	0.18 ± 0.01	94.29 ± 1.17	0.21 ± 0.01	124.75 ± 6.21	0.34 ± 0.04
**5kDP5-IONP**	59.42 ± 0.22	0.11 ± 0.01	60.74 ± 0.51	0.13 ± 0.02	57.52 ± 0.94	0.07 ± 0.01
**5kDP10-IONP**	57.59 ± 0.69	0.11 ± 0.01	60.88 ± 0.58	0.09 ± 0.01	60.64 ± 1.11	0.10 ± 0.01

Table S2. Summary of TEM and Zeta Potential.

IONP coatings	TEM		Surface charge (day 1)
	Core size (day 1) [nm]	Core size (day 32) [nm]	ζ potential [mV]
**5kDP5-IONP**	26.28 ± 3.17	28.49 ± 3.36	-15.00 ± 7.48
**5kDP10-IONP**	26.90 ± 3.01	26.72 ± 2.81	-25.20 ± 7.50

Table S3. Summary of calculations of Flory radius (R_F), grafting distance (D) and thickness of PEG layer (L) and prediction of PEG conformation.

Structure of the shell	σ [chains nm^{-2}]	R_F (nm)	D [nm]	L [nm]	R_F/D	PEG conformation
PEG5k-AsAm$_5$	1.222	5.969	1.02	19.38	3.40	Brush
PEG5k-AsAm$_{10}$	0.727	5.969	1.324	16.29	2.86	Brush

EQUATIONS

Estimation of PEG conformation

Equation S1. Mathematic equation to calculate Flory radius (R_F), grafting distance (D) and thickness of PEG layer (L).[1,2]

$$R_F = \alpha N^{3/5} \quad (1)$$

$$D = 2(A/3.14)^{1/2} \quad (2)$$

$$L = N(\alpha^{5/3})/D^{2/3} \quad (3)$$

where α is the monomer length (0.35 nm for PEG), N is the number of PEG repeating units, A is the area occupied per PEG chain. A is calculated from the measured grafting density (chains per nm^2) by using equation A= 1/density.

For 5kDP5-IONP (PEG5k-AsAm$_5$@IONPs)

α = 0. 35nm, N = 113

σ = 1.222 => A = 0.8183 nm^2

=> R_F = 5.969nm, D = 1.02nm, L = 19.38 nm

For 5kDP10-IONP (PEG5k-AsAm$_{10}$@IONPs)

α = 0. 35nm, N = 113

σ = 0.727 => A = 1.376 nm^2

=> R_F = 5.969nm, D = 1.324nm, L = 16.29 nm

REFERENCES

1. de Gennes, P.G. Conformations of Polymers Attached to an Interface. *Macromolecules* **13**, 1069–1075 (1980).
2. Li, M., *et al.* Brush Conformation of Polyethylene Glycol Determines the Stealth Effect of Nanocarriers in the Low Protein Adsorption Regime. *Nano Letters* **21**, 1591–1598 (2021).
